# Design of a multicentre randomized trial to evaluate CT colonography versus colonoscopy or barium enema for diagnosis of colonic cancer in older symptomatic patients: The SIGGAR study

**DOI:** 10.1186/1745-6215-8-32

**Published:** 2007-10-27

**Authors:** Steve Halligan, Richard J Lilford, Jane Wardle, Dion Morton, Pauline Rogers, Katherine Wooldrage, Rob Edwards, Reshma Kanani, Urvi Shah, Wendy Atkin

**Affiliations:** 1 Department of Specialist Radiology, University College Hospital London, Euston Road, London; 2 Department of Public Health, University of Birmingham, Egbaston, Birmingham; 3 Health Behaviour Research Centre, University College London, Gower Street, London; 4 Department of Surgery, Queen Elizabeth Hospital, Egbaston, Birmingham; 5 Cancer Research UK Colorectal Cancer Unit, St. Mark's Hospital, Harrow

## Abstract

**Background and Aims:**

The standard whole-colon tests used to investigate patients with symptoms of colorectal cancer are barium enema and colonoscopy. Colonoscopy is the reference test but is technically difficult, resource intensive, and associated with adverse events, especially in the elderly. Barium enema is safer but has reduced sensitivity for cancer. CT colonography ("virtual colonoscopy") is a newer alternative that may combine high sensitivity for cancer with safety and patient acceptability. The SIGGAR trial aims to determine the diagnostic efficacy, acceptability, and economic costs associated with this new technology.

**Methods:**

The SIGGAR trial is a multi-centre randomised comparison of CT colonography versus standard investigation (barium enema or colonoscopy), the latter determined by individual clinician preference. Diagnostic efficacy for colorectal cancer and colonic polyps measuring 1 cm or larger will be determined, as will the physical and psychological morbidity associated with each diagnostic test, the latter via questionnaires developed from qualitative interviews. The economic costs of making or excluding a diagnosis will be determined for each diagnostic test and information from the trial and other data from the literature will be used to populate models framed to summarise the health effects and costs of alternative approaches to detection of significant colonic neoplasia in patients of different ages, prior risks and preferences. This analysis will focus particularly on the frequency, clinical relevance, costs, and psychological and physical morbidity associated with detection of extracolonic lesions by CT colonography.

**Results:**

Recruitment commenced in March 2004 and at the time of writing (July 2007) 5025 patients have been randomised. A lower than expected prevalence of end-points in the barium enema sub-trial has caused an increase in sample size. In addition to the study protocol, we describe our approach to recruitment, notably the benefits of extensive piloting, the use of a sham-randomisation procedure, which was employed to determine whether centres interested in participating were likely to be effective in practice, and the provision of funding for dedicated sessions for a research nurse at each centre to devote specifically to this trial.

**Trial registration:**

Current Controlled Trials ISRCTN95152621

## Background

Computed tomographic colonography (CTC) is a relatively novel health technology used to examine the large bowel. Specifically, it combines helical CT scanning of the cleansed and distended colorectum with complex image rendering techniques, and is used primarily to detect colorectal neoplasia. CTC was first described in 1994 [1] and has been evaluated by several comparisons with colonoscopy, both single-centre [2,3] and multi-centre [4,5]. Meta-analysis of studies where patients have both CTC and colonoscopy suggests that CTC has high average sensitivity and specificity for diagnosis of colorectal neoplasia [6,7]. There are no randomised studies. Radiologists have repeatedly stressed a role in colorectal cancer screening, with claims that CTC combines excellent sensitivity for adenomatous polyps (the precursor of colorectal cancer) with safety and acceptability [8]. However, at the time of writing only a single published study has examined a representative sample [9] and the overwhelming majority of studies have been exclusively based on patients with symptoms suggestive of cancer. While these studies have made a case for screening by focussing on the detection of polyps, in reality polyps in these patients are serendipitous findings and not responsible for symptoms. The emphasis on polyps has obscured evidence that the sensitivity of CTC for colorectal cancer is possibly as high as colonoscopy and likely higher than barium enema, the standard tests currently used for diagnosis. A recent meta-analysis found an average sensitivity for cancer of 96% (95%CI, 91% to 99%) [6]. It follows that the role of CTC for diagnosis of symptomatic cancer deserves more attention.

Around 363,000 cases of colorectal cancer occur each year in the European Union and 148,000 in the USA [10]. It is important to make an early diagnosis because prognosis depends on tumour stage but local variations in clinical practice can delay this [11]. English patients with symptoms suggestive of colorectal cancer must be now be seen by a specialist within 2-weeks of referral by their general practitioner [12]. A "whole-colon" test is frequently requested to search for cancer but because the "suggestive symptoms" are non-specific and common in the general population (e.g. abdominal pain, change in bowel habit), most patients investigated do not have the disease. Colonoscopy is the most sensitive test but is expensive, resource intensive, technically difficult, and has a small but well-recognised morbidity and even mortality [13]. Furthermore, most patients investigated are elderly, the group most at risk from adverse events. Barium enema is safer than colonoscopy [14] but has reduced sensitivity [15], and interpretation skills are declining [16]. CTC is safer than colonoscopy [17,18] while being more sensitive than barium enema, and appears to be more acceptable to patients than either of the other tests [8]. CTC may also be performed by technicians and in principle the data could be read by computer-assistance [19], accelerating diagnosis and throughput.

The paragraphs above make the case that CTC may combine accurate diagnosis of colorectal cancer with safety and efficiency but CTC also presents the opportunity to investigate other intra-abdominal organs (unlike colonoscopy and barium enema) because these are included on the scan. Pathology outside the colon may sometimes underpin patients' symptoms and it is possible that CTC could have more diagnostic utility overall than tests restricted to the colorectum. However, characterisation of extra-colonic findings may also consume significant health-care resources investigating 'abnormalities' that ultimately prove insignificant: A recent study found that 116 (52%) of 225 symptomatic patients studied by CTC had one or more extracolonic findings, consuming £34,329 to pursue – a figure exceeding the original costs of CTC and effectively doubling investigation costs overall [20]. It is unknown how these costs, along with inconvenience, anxiety, morbidity and occasionally even mortality, are offset by the benefit to those with sub-clinical but potentially lethal diseases. CTC will also serendipitously detect significant adenomatous polyps (defined as 1 cm or lager in size) that are not responsible for symptoms but which, having been detected, will need endoscopic removal.

In this article we describe the protocol for the SIGGAR trial, a randomised comparison of CTC against either colonoscopy or barium enema. Participating radiologists are members of SIGGAR, the Royal College of Radiologists Special Interest Group in Gastrointestinal and Abdominal Radiology (now BSGAR, British Society of Gastrointestinal and Abdominal Radiology [21]). Research on this topic was commissioned by the UK Health Technology Assessment (HTA) programme in 2002, who are funding the study (proposal 02/02/01).

### Study objectives

• To compare the detection rates and diagnostic sensitivity for significant colorectal neoplasia of CTC versus barium enema and CTC versus colonoscopy.

• To examine psychological and physical morbidity associated with each diagnostic pathway.

• To determine costs of diagnosis associated with each diagnostic pathway.

• To determine the frequency, clinical relevance, economic costs, and psychological and physical morbidity associated with detection of extracolonic lesions by CTC.

• To use data accumulated from the trial to populate models framed to summarise the health effects and costs of alternative approaches to detection of significant colonic neoplasia in patients of different ages, prior risks and preferences.

Diagnostic pathway is defined by the procedure to which the patient is initially randomised, recognising that some patients will undergo more than one of the tests ('conversion'), for example due to technical failure or to confirm the result of the initial test.

## Design and methods

### General

**Figure 1:**
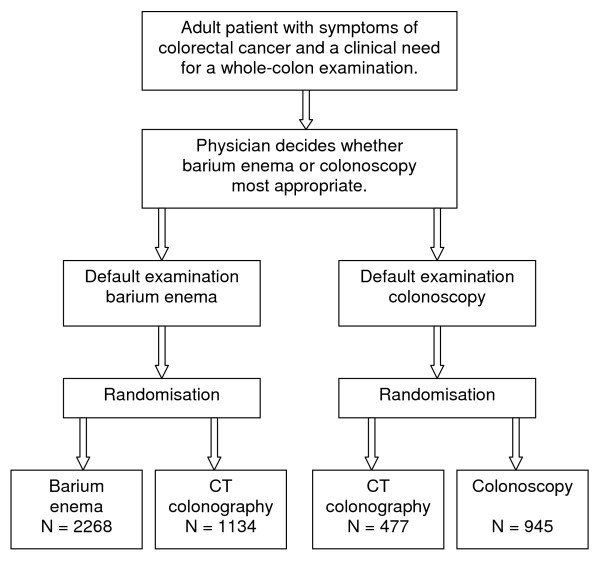
Flow chart of patient recruitment to each sub-trial with sample size for each group randomised.

The SIGGAR trial comprises two parallel prospective randomised multicentre studies, each with a 2:1 randomisation in favour of the 'default' whole-colon examination (Figure 1). One sub-trial compares CTC against colonoscopy and the other CTC against barium enema. Recruitment commenced April 2004.

### Inclusion/exclusion criteria

Eligible patients are aged 55 years or older, able to give informed consent, with symptoms or signs considered suggestive of colorectal cancer by the referring physician (e.g. change in bowel habit, rectal bleeding, abdominal pain, anaemia). A decision to perform a "whole-colon" examination to diagnose/exclude colorectal cancer will have been taken by the clinician-in-charge and patients must therefore be fit to undergo full bowel preparation. Patients with a known genetic predisposition to cancer, inflammatory bowel disease, or who are being followed-up for colorectal cancer are excluded. They must have had no "whole-colon" examination in the past six months.

Participating centres must have an established and efficient fast-track clinical referral system for managing patients referred with symptoms suggestive of colorectal cancer, with a throughput sufficient to recruit at least 18 patients a month. Centres must also be able to satisfy technical stipulations for the diagnostic interventions (see section below). Participating centres were chosen fro those interested in participating via a sham-randomisation procedure (see Discussion).

### Ethical arrangements and consent

The SIGGAR trial achieved multi-centre ethical committee (MREC) approval in January 2004 and is being conducted in accordance with Medical Research Council guidelines for good clinical practice in clinical trials and the Research Governance Framework. Informed consent is a prerequisite. The trial is supervised by an independent data monitoring committee (DMC) and trial steering committee (TSC) (see Acknowledgements).

### Randomisation

Basic demographic and clinical information is collected and patients registered at the time they are judged potentially eligible for randomisation, usually by inspection of referral forms/clinic letters in the days just prior to attendance. Eligible, registered patients are then randomised after agreement by the lead clinician and patient but data relating to registered patients who are ultimately not randomised are retained along with the reason for non-randomisation, so that external validity can be judged and estimates of generalisability made once the data are known.

Randomisation is performed centrally, at the trial office. Patients are randomised 2:1 in favour of the default diagnostic test (i.e. colonoscopy or barium enema). On the advice of the DMC, details of stratification/blocks are confidential since it is theoretically possible to predict allocation for some patients if past allocation and block site is known.

### Diagnostic interventions

#### 1. CT colonography

CT colonography has undergone rapid technological evolution since first described, which continues at the time of writing. There have been particular advances in visualisation software. Because recruitment will take several years, the protocol allows investigators to adopt advances during the course of the trial so that findings are as up to data as possible. The protocol for CTC is based on expert consensus where this exists [22]. For example, multi-detector row scanners are mandatory and patients must undergo full bowel preparation, and both prone and supine scanning at a minimum collimation of 2.5 mm. Where no consensus exists, for example administration of intravenous contrast and spasmolytic [23], local investigators have freedom to choose in line with their personal preference, which reflects normal clinical practice and enhances generalisability. Interpretation software has been provided (Voxar, Barco, Anderson Place, Edinburgh, United Kingdom, and V3D, Viatronix Inc, Stony Brook, NY), but local investigators are free to use other commercially available platforms according to their preference. Observers may use 2-D or 3-D reading, or any combination of the two, reflecting individual preference in normal clinical practice. Observers may report any colonic or extra-colonic finding that they feel may explain patients' symptoms or which they consider potentially relevant, in line with personal diagnostic thresholds. Interpretation is limited to consultant radiologists with a demonstrable subspecialist interest in gastrointestinal imaging (those attending the gastrointestinal multi-disciplinary team meetings for example), reflecting established practice for investigation of symptomatic patients. All have previous experience of CTC that has been supplemented with a two-day training course for the purposes of the study. Trial centres encompass both teaching and district general hospitals to enhance generalisability.

#### 2. Barium enema

Barium enema examinations are performed after full bowel preparation, using best current practice; i.e. digital fluoroscopy (512 matrix minimum) of the double-contrasted colorectum to the caecum, supplemented by overcouch decubitus films. A spasmolytic and carbon dioxide is used. Examinations are performed by either radiologists with a subspecialty interest in gastrointestinal radiology or radiographic technicians fully trained in barium enema procedures, reflecting conventional practice. Enemas are reported by radiologists with a subspecialty interest in gastrointestinal radiology.

#### 3. Colonoscopy

Colonoscopy is performed by experienced colonoscopists, either physicians or surgeons who have satisfied stipulations on training made by the joint advisory group on gastrointestinal endoscopy (JAG) [24]. Modern video-endoscopes are used and sedation and analgesia administered when judged clinically necessary in each individual case. Colonoscopy to the caecum is intended initially in all cases.

Datasheets for each diagnostic procedure are completed by practitioners and detail clinical findings, procedural information (quality of bowel preparation, completeness, difficulty, diagnostic confidence, for example), any adverse events, and data necessary to calculate resource consumption subsequently.

We will obtain data for diagnosis of colorectal cancer for randomised patients from the Office for National Statistics (ONS) so that we have a denominator for calculation of sensitivity in addition to detection rates by each diagnostic test.

### Outcome measures

#### Primary outcome measures

1. **CTC vs barium enema**. Detection rates and diagnostic sensitivity of significant colonic neoplasia by each test

2. **CTC vs colonoscopy**. Requirement for additional tests needed to diagnose or exclude significant neoplasia.

Significant colorectal neoplasia is defined as cancer or polyps 1 cm or larger. Such polyps may have a clinical consequence for patients even if they are not the cause of symptoms. We do not focus on detection rates in the colonoscopy sub-trial because available evidence suggests that colonoscopy and CT have equivalent sensitivity for significant colorectal neoplasia.

#### Secondary outcome measures

• Time to diagnosis or exclusion of cancer for each diagnostic pathway. Time to diagnosis of pathology, if any, responsible for the presenting symptoms.

• Frequency and nature of any adverse events split by diagnostic pathway.

• Technical adequacy for the different diagnostic procedures; need for repeat procedures.

• Psychological reactions, adverse events, and preferences for each diagnostic test. The precise outcomes to be measured have been determined by a qualitative pilot study. Interactions with collected demographic data (e.g. age, gender, socio-economic status) and procedures (e.g. number of procedures) will be assessed.

• Economic costs associated with different investigative trajectories. Patient specific records of costs and outcomes including the influence of conversion to other tests and multiple investigations. Cost utility models to compare management plans with outcome cost will be defined. Test characteristics for CTC and patient preferences/quality of life measures will be combined with the economic evaluation in order to determine overall cost utility.

• Modelling: A generic model will be developed to determine total effectiveness for each diagnostic pathway, populated by outcomes from the trial data so that the findings can be extrapolated beyond the trial. The model will pay particular attention to the incidence, relevance, psychological reaction to, and costs of incidental extra-colonic lesions detected by CTC.

### Sample size

The initial sample size was calculated using the following assumptions:

• Randomisation will be 2:1 in favour of the default diagnostic test (i.e. colonoscopy or barium enema).

• The prevalence of significant colorectal neoplasia (i.e. cancer or polyps 1 cm or larger) in symptomatic adults is approximately 15%.

• In each diagnostic pathway, additional examinations will inevitably be required to diagnose or exclude cancer in some patients. We assumed that the proportions requiring further examinations are respectively 14% for colonoscopy (based on anticipated completion rates), 20% for CTC (based on equivocal findings needing further investigation, the need for polypectomy or biopsy in those patients with neoplasia, and also those with significant extracolonic findings needing further investigation), and 15% for barium enema (based on need for further investigation in those with polyps, false positive findings, and incomplete examination in an elderly group, e.g due to incontinence).

#### 1. Diagnostic yield: CTC vs barium enema

The original sample size assumed a detection rate of significant neoplasia of 15% for CTC and 10% for barium enema. With a 2:1 randomisation in favour of barium enema and an alpha of 0.05 (two-tailed), 2160 patients gave us 90% power to detect a difference in detection rates. A revised sample size was needed once the trial was progressing as the above assumptions were found to be overestimates (see Discussion). The revised sample size assumed a detection rate of 7.5% for CTC and 5% for barium enema. To achieve 80% power to detect a difference in detection rates with 2:1 randomisation in favour of barium enema and an alpha of 0.05 (two-tailed), 3402 patients were needed.

#### 2. Subsequent colonic investigations: CTC vs colonoscopy

The available literature suggests that CTC and colonoscopy are equivalent for diagnosis of cancer. A prevalence of significant abnormality of only 15% means that a trial at least ten times larger would be required to detect a diagnostic difference with reasonable power. In any event, because such differences are likely to be small, the role of CTC will instead turn on organisational factors, economic costs, and on the personal reactions of patients. The number of patients who need further investigations before a diagnosis is made or refuted is central to this; the "conversion rate". All the interventions tested will sometimes incur other tests, because of technical failure for example, but the conversion rate is likely to be highest with CTC because more structures are visualised than with colonoscopy or barium enema.

Conversion rates of 20% for CTC and 14% for colonoscopy were assumed. Assuming 2:1 randomisation in favour of colonoscopy and an alpha of 0.05 (two-tailed), an original sample size of 2160 provided 90% power to detect a difference in conversion rates. As the trial progressed, it became apparent that recruitment to this sub-trial was lower than anticipated. Power was lowered to the more conventional 80% and retaining other assumptions as above, the revised sample size was 1430.

Power calculations for psychological outcomes were based on the Short form of the State Trait Anxiety Scale (STAI). 4320 patients randomised overall (see sections above) gave considerable overall power. However, our findings need to be particularised to different settings and subgroups so that the modelling exercise is accurate. We therefore need adequate power in the smallest important patient group – i.e. those needing further investigations following CTC, including those with a positive diagnosis of cancer (i.e. 20% of 720 + 720 = 288). This is sufficient to give us 90% power to detect an 0.5 s.d. difference in STAI scores whose mean value is 10.1 (s.d. 3.5), allowing for a 20% questionnaire non-completion rate.

### Analysis

The two sub-trials will be analysed as two separate case-control studies. In each sub-trial, cases and controls will be compared regarding covariates, including age, sex and centre, using chi-squared or Fisher's exact test for categorical variables and an unpaired t-test for continuous variables. The primary outcomes will be analysed using multivariable logistic regression with study group as the primary explanatory variable and adjustment for other covariates, including age, sex and centre. Centre will be considered as a random variable and the analysis will be clustered on centre. Secondary outcomes will be analysed using poisson regression for counts and logistic regression for proportions with the same explanatory variables. The data will be analysed in Stata 9.2 and variables will be considered significant at the 5% level.

## Discussion

### Recruitment

At the time of writing (July 2007) 5025 patients have been randomised; 3562 to the barium enema sub-trial and 1463 to the colonoscopy subtrial. Recruitment to the SIGGAR trial has gone well at a time when large randomised trials of diagnostic interventions are finding it difficult to reach their targets. Also, the trial does not obey the anecdotal "80:20 rule", which states that 80% of the work is usually done at 20% of the centres; most of our centres are recruiting well. We believe that there are several factors that underpin this success, which others considering implementing such a trial may find useful. Potential participating centres were identified via expressions of interest solicited through a UK-wide network of subspecialist gastrointestinal radiologists (SIGGAR). This introduced a spectrum bias towards interested subspecialists, who may be more likely to participate actively in research. We had identified CT scanner capacity as the main constraint on patient accrual. Interested sites were therefore asked to indicate potential CT capacity for the trial so that estimates of the number of possible CT scans per year were evidence-based, and powering was pragmatic. In particular, sample size can be achieved by either increasing the number of centres or by more intense and prolonged recruitment in fewer centres. We analysed the consequences for participating hospitals in terms of 're-engineering' their services for the trial in order to arrive at a balance between the number of participating centres and their marginal cost, arriving initially at the figure of 10 centres recruiting for 2-years.

Interested centres were also asked to perform a "sham-randomisation" exercise both to prove that they could identify potential patients in sufficient numbers, and to focus their attention on how this might be achieved. Over a two-month period each centre was asked to identify patients who satisfied trial inclusion criteria, record simple demographics for them (age, sex, symptoms, referral route, and whole-colon investigation requested), and to enter these on a secure, password-protected web-based database. No patients were approached directly but this "sham-randomisation" provided an evidence-based estimate of how each centre might perform in reality, once the trial was running. 25 centres registered for the sham randomisation and the 10 best-performing were ultimately selected (four of these had already been identified as pilot sites because of extensive pre-existing familiarity with CTC). We believe this procedure restricted participation to those centres most likely to recruit well in practice and identified others in advance who would probably fare poorly, thereby avoiding resource wastage. For example, some centres who registered for the sham-randomisation ultimately failed to register a single patient.

Trial procedures were also piloted extensively at a single centre – 76 patients were randomised and a further 71 registered over 5 months before recruitment was rolled-out to the centres selected by the sham-randomisation. Piloting identified efficient recruitment routes (for example, by targeting outpatient clinics receiving most referrals), tested trial materials, and allowed problems to be identified and solved at a single centre so that others joining subsequently could benefit from procedures that had already been proven to work in practice. For example, we were able to develop strategies for dealing with participants who strongly desired allocation to CT colonography, a problem we had not anticipated in advance. Roll-out was implemented in a step-wise fashion, rather than starting all other centres simultaneously. This allowed the trial manager to concentrate on one or two new centres at a time, and reduced the strain on the trial-office. All 10 centres had started recruiting by May 2005, approximately one-year after the first pilot site. Other factors contributing to success have been the provision of funding sufficient to purchase research sessions dedicated to the trial at each centre, rather than relying upon the research nurses who have to manage a portfolio of trials. We have also hosted several investigator/collaborator meetings which provide occasions for the principal investigators to praise and motivate the centres. The meetings are also an excellent opportunity for local investigators to share problems and solutions in an informal setting.

### Sample size assumptions

It is important to recognise that in a trial such as SIGGAR, patients do not contribute to the primary end-point unless they ultimately prove to have the target disease. Centres with a low prevalence of cancer are therefore not as effective as their recruitment suggests at first glance: It is well recognised that the prevalence of disease in trials is lower than would be expected normally. In such a situation it is important that the investigators and Data Monitoring Committee (DMC) continually reassess recruitment in the light of the prevalence of abnormality. An interim analysis carried out for the Trial Steering Committee Meeting in December 2005, at a time when 1237 patients had been randomised, noted that the prevalence of abnormality was 7% overall, compared with the 10% expected prior to the trial starting. Thus the trial had accrued fewer end-points than anticipated. The prevalence of abnormality also varied between sub-trials, being 12% in the colonoscopy sub-trial but only 5% in the barium enema sub-trial. Furthermore, although we had anticipated a 50:50 split between the two sub-trials, 862 (69.7%) had been recruited to the barium enema sub-trial (where the prevalence of abnormality is lowest), and only 375 to the colonoscopy sub-trial. A request for extension was made successfully, pointing out the need to increase the numbers in the barium enema sub-trial above the original sample size estimate while also increasing the rate of recruitment to the colonoscopy sub-trial. Power was also reduced from 90% to the more conventional 80% overall. The original sample size of 4320 (2160 randomised in each sub-trial) is now 4832, with 3402 patients needed in the barium enema sub-trial and 1430 in the colonoscopy sub-trial to acquire sufficient end-points, taking into account the difference in disease prevalence between the two sub-trials. At the time of writing 5025 patients have been recruited and the trial is in a position to close assuming that sufficient end-points have been accrued.

One centre withdrew in 2005 following the recommendation of the Data Monitoring Committee. At the same time a decision was made to extend recruitment to other centres in order to accelerate recruitment (see Appendix). Increasing the number of centres was feasible because trial procedures and materials had already been developed and piloted, rolled-out, and were running efficiently at most centres. Also, the marginal cost of a new centre was low versus the cost of the trial as a whole. The decision to implement new centres was taken only when effective recruitment was established at existing centres; new centres would be very unlikely to enhance a trial that is failing to recruit at existing centres. It is also vital to keep existing centres motivated and ensure that the data are forthcoming. We have exerted considerable effort on retrieving completed data forms from centres.

## Conclusion

The SIGGAR trial is a multi-centre randomised comparison of CT colonography against either barium enema or colonoscopy. It aims to determine the diagnostic yield of each test for significant colorectal neoplasia and to quantify other aspects of diagnosis including patient acceptability, adverse events, and costs. Excellent recruitment to the SIGGAR trial to date demonstrates the benefits of a sham-randomisation procedure used to select effective centres, extensive piloting of materials and procedures, and staged implementation of participating centres.

## Abbreviations

CTC: Computed tomographic colonography.

DMC: Data monitoring committee.

## Competing interests

SH receives research support for CT colonography studies from Medicsight plc, Barco, and Viatronix.

RJL, JW, DM, PR, KW, RE, RK, US, WA declare no competing interests.

## Authors' contributions

SH, RJL, JW, DM, RE, WA conceived and designed the study, and obtained funding. RK managed recruitment. RE and US designed the database. PR, KW, RK, US collated data and performed interim analysis. SH drafted the manuscript with help from all authors. All authors read and approved the final manuscript.
